# Comparative Analysis of the Effects of Olive Oil Hydroxytyrosol and Its 5-*S*-Lipoyl Conjugate in Protecting Human Erythrocytes from Mercury Toxicity

**DOI:** 10.1155/2018/9042192

**Published:** 2018-04-12

**Authors:** Arbace Officioso, Lucia Panzella, Fabiana Tortora, Maria Laura Alfieri, Alessandra Napolitano, Caterina Manna

**Affiliations:** ^1^Department of Biochemistry, Biophysics and General Pathology, University of Campania “Luigi Vanvitelli”, Naples, Italy; ^2^Department of Chemical Sciences, University of Naples “Federico II”, Naples, Italy

## Abstract

Oxidative stress is one of the underlying mechanisms of the toxic effects exerted by mercury (Hg) on human health. Several antioxidant compounds, including the olive oil phenol hydroxytyrosol (HT), were investigated for their protective action. Recently, we have reported that 5-*S*-lipoylhydroxytyrosol (Lipo-HT) has shown increased antioxidant activities compared to HT and exerted potent protective effects against reactive oxygen species (ROS) generation and oxidative damage in human hepatocellular carcinoma HepG2 cell lines. In this study, the effects of Lipo-HT and HT on oxidative alterations of human erythrocytes induced by exposure to 40 *μ*M HgCl_2_ were comparatively evaluated. When administered to the cells, Lipo-HT (5–20 *μ*M) proved nontoxic and it decreased the Hg-induced generation of ROS, the hemolysis, and the depletion of intracellular GSH levels. At all tested concentrations, Lipo-HT exhibited higher ability to counteract Hg-induced cytotoxicity compared to HT. Model studies indicated the formation of a mercury complex at the SH group of Lipo-HT followed by a redox reaction that would spare intracellular GSH. Thus, the enhanced erythrocyte protective action of Lipo-HT from Hg-induced damage with respect to HT is likely due to an effective chelating and reducing ability toward mercury ions. These findings encourage the use of Lipo-HT in nutraceutical strategies to contrast heavy metal toxicity in humans.

## 1. Introduction

Hydroxytyrosol (HT) is a dietary phenolic antioxidant compound, naturally present in virgin olive oil, contributing to its great oxidative stability and prolonged shelf life [1]. HT is endowed with several pharmacological properties [2–4], including anti-inflammatory [5], antiatherogenic [6], and anticancer activities [7]. Mechanisms underlying HT biological effects include both radical scavenging properties and metal chelating activity [8–11]. Cytoprotective effects of HT against xenobiotic compounds have been reported, including protection from acrolein-induced DNA damage [12], acrylamide-induced mitochondrial dysfunction [13], and carbon tetrachloride-induced oxidative stress [14].

Mercury (Hg) is a highly toxic, redox-active heavy metal which represents one of the main agents responsible for environmental pollution [15, 16]. The health consequences of human exposure to Hg can be severe [17, 18] and include renal injury [19] and neuronal disorders [20]. In this respect, Hg is considered a potential contributing factor to Alzheimer's and Parkinson's diseases [21]. Red blood cells (RBC) may also represent an important target of Hg toxicity. This metal, indeed, preferentially accumulates in these cells and induces morphological changes which increase their procoagulant activity [22–24]. Finally, an increasing body of data suggests a positive correlation between Hg exposure and the onset of cardiovascular diseases [25].

The increased formation of reactive oxygen species (ROS) is generally agreed to be one of the key mechanisms responsible for Hg-induced toxicity [26–28]. Mercury is endowed with high affinity for sulfhydryl groups, and it is therefore able to react with low-molecular-weight thiols, including glutathione (GSH) [29, 30].

Indeed, the cellular levels of GSH have been shown to significantly decrease following Hg exposure, with consequent impairment of the antioxidant defence system [31]. Following the oxidative stress hypothesis of Hg toxicity, a number of antioxidants [32, 33], including phenolic compounds [34–36], have been proposed and tested for their protective action. In this connection, we recently provided experimental evidence that HT has the potential to modulate cytotoxicity and the oxidative stress induced in human RBC by Hg treatment [37, 38]. Using the scanning electron microscopy technique, we also showed that HT treatment significantly reduces echinocyte formation [37], a morphological RBC alteration reported to correlate with increased coagulability of these cells [39]. Furthermore, a recent study by Mohan et al. highlighted the efficacy of HT in preventing methylmercury-induced genotoxicity and apoptosis in IMR-32 human neuroblastoma cells. HT was shown to inhibit methylmercury-induced neuronal cell dysfunction as highlighted by the decrease in ROS formation and maintenance of an efficient endogenous defence system, including GSH levels and superoxide dismutase and catalase activities [40].

**Figure 1:**
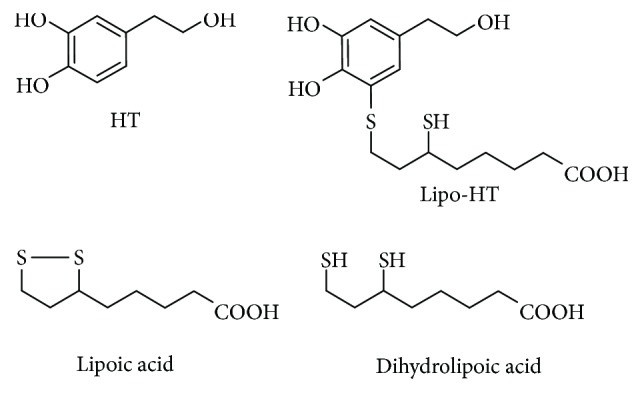


In the last few years, several HT derivatives with enhanced antioxidant and pharmacological properties have been described [41–46]. Among these, 5-S-lipoylhydroxytyrosol (Lipo-HT), synthesized by conjugation of HT with the biologically relevant thiol dihydrolipoic acid, showed increased antioxidant activities compared to HT in several chemical assays and exerted potent protective effects against ROS generation and oxidative cell damage in human hepatocellular carcinoma HepG2 cell lines (Figure 1) [47, 48]. Insertion of the sulfur-containing chain was found to exert a crucial influence on the reactivity of the adjacent catechol system conferring a more pronounced lipophilic character on the core system and resulting in an overall potentiation of the antioxidant activity of HT. This effect was also demonstrated in the case of other naturally occurring catechols [49, 50]. Because of the peculiar combination in its molecular scaffold of a number of redox-active groups, Lipo-HT exerts a multidefence antioxidant action through different mechanisms, including H-atom release, ferric and cupric ion reduction, and OH radical scavenging [48].

The aim of this study was to test the ability of Lipo-HT to prevent human RBC from the oxidative alterations induced by Hg treatment in comparison with the parent HT. These cells are a unique cellular model for *in vitro* studies which investigate oxidative stress-related alterations as well as Hg toxicity [23, 24, 37, 38]. Mercury(II)chloride (HgCl_2_) was selected for running the experiments since mercury is a well-known oxidative stress inducer [51].

The rationale of testing Lipo-HT in this toxicity model system stems from the possibility of exploiting, in addition to the antioxidant power of the catechol moiety activated by the adjacent thioalkyl group, the free SH group of dihydrolipoic acids capable of effectively chelating Hg. Cytoprotective effects were indeed observed in the case of this conjugate, whose mechanisms were initially investigated at the molecular level by model experiments.

## 2. Materials and Methods

### 2.1. Chemicals

2′,7′-Dichlorodihydrofluorescein diacetate (DCFH-DA), mercuric chloride (HgCl_2_), tyrosol, lipoic acid, 5,5′-dithiobis(2-nitrobenzoic acid) (DTNB) (Ellman's reagent), and HT were from Sigma Chemical Co. All other chemicals used were of the highest purity grade available.

### 2.2. Methods

UV/Vis spectra were recorded on a Jasco V-730 spectrophotometer. HPLC analysis was carried out on an Agilent instrument equipped with a UV detector set at 254 nm. The chromatographic separation was achieved on a Phenomenex SphereClone ODS column (250 mm × 4.6 mm, 5 *μ*m) using binary gradient elution conditions as follows: 0.1% trifluoroacetic acid (solvent A) and acetonitrile (solvent B), from 5% to 90% B, 0–45 min, and flow rate 0.7 mL/min. LC/MS analyses were run on a LC/MS ESI-TOF 1260/6230DA Agilent instrument operating in positive ionization mode in the following conditions: nebulizer pressure 35 psig; drying gas (nitrogen) 8 L/min, 325°C; capillary voltage 3500 V; and fragmentor voltage 175 V. An Eclipse Plus C18 column, 150 × 4.6 mm, 5 *μ*m, at a flow rate of 0.4 mL/min was used, with the same mobile phase as above.

### 2.3. Synthesis of 5-*S*-Lipoylhydroxytyrosol (Lipo-HT)

The procedure previously described was adopted [48]. The reaction was carried out on tyrosol (1.0 g) affording Lipo-HT in about 30% yield. The purity of the compound was evaluated (>95%) by HPLC and ^1^H NMR analysis.

### 2.4. Preparation of RBC and Treatment with Hg

The RBC fraction was obtained from whole blood obtained from human healthy volunteers deprived of leucocytes and platelets by filtration on a nylon net, washed twice with isotonic saline solution (0.9% NaCl), and finally resuspended with buffer A (5 mM Tris-HCl containing 0.9% NaCl, 1 mM MgCl_2_, and 2.8 mM glucose, pH 7.4) to obtain a 10% hematocrit. Intact RBC were incubated at 37°C with 40 *μ*M HgCl_2_ for 4 h. For the experiments with Lipo-HT or HT, stock solutions (100 mM) were prepared in DMSO. Just before the experiments, these solutions were diluted to 1 mM with the isotonic saline solution and added to the incubation medium to obtain the desired concentration 5 min before addition of Hg. As a control, the effects of the highest volume of DMSO used on RBC in the presence of Hg were also evaluated and found to be negligible. RBC from each donor were used for a single assay in triplicate. Each experiment was repeated on RBC obtained from three different donors.

### 2.5. Determination of Hemolysis

The extent of hemolysis was determined spectrophotometrically, according to Tagliafierro et al. [37]. At the end of the incubation, the reaction mixture was centrifuged at 1100*g* for 5 min and the hemoglobin (Hb) released was evaluated by measuring the absorption of the supernatant at 540 nm. Packed RBC were hemolyzed with ice-cold distilled water at 40 : 1 *v*/*v*, the lysate was centrifuged at 1500*g* for 10 minutes, and the supernatant (B) was absorbed at 540 nm. The percentage of hemolysis was calculated as the ratio of the readings (A/B) × 100.

### 2.6. Determination of ROS

To quantify ROS generation, the dichlorofluorescein (DCF) assay was used according to Tagliafierro et al. [37]. Intact RBC were incubated with DCFH-DA at 10 *μ*M final concentration for 15 min at 37°C. After centrifugation at 1200*g* for 5 min, the supernatant was removed and the hematocrit value was adjusted to 10% with buffer A. RBC were then treated with HgCl_2_ in the dark. At the end of incubation, 20 *μ*L of RBC were diluted in 2 mL of water and the fluorescence intensity of the oxidized derivative DCF was recorded (*λ*_ex_ 502 nm; *λ*_em_ 520 nm). The results were expressed as fluorescent intensity/mg of Hb.

### 2.7. Quantification of Intracellular GSH

The intracellular GSH content was determined spectrophotometrically by reaction with DTNB reagent, according to van den Berg et al. [52]. The samples (0.25 mL) described above were centrifuged, the supernatants were removed, and RBC were lysed by the addition of 0.6 mL of ice-cold water. Proteins were precipitated by the addition of 0.6 mL ice-cold metaphosphoric acid solution (1.67 g metaphosphoric acid, 0.2 g EDTA, and 30 g NaCl in 100 mL of water). After incubation at 4°C for 5 min, the protein precipitate was removed by centrifugation at 18000*g* for 10 min and 0.45 mL of the supernatant was mixed with an equal volume of 0.3 M Na_2_HPO_4_. 100 *μ*L of DTNB solution (20 mg DTNB plus 1% of sodium citrate in 100 mL of water) was then added to the sample, and after a 10 min incubation at room temperature, the absorbance of the sample was read against the blank at 412 nm.

### 2.8. Reaction of Lipo-HT or HT with Hg^2+^ Ions

5 *μ*L of a 100 mM HgCl_2_ solution was added to 5 mL of buffer (pH 7.4), followed by 5 *μ*L of a 50 mM DMSO solution of Lipo-HT or HT (100 *μ*M and 50 *μ*M final concentrations for Hg^2+^ and Lipo-HT or HT, resp.). The reaction mixture was taken under stirring and periodically analyzed by HPLC, LC/MS, and UV/Vis spectroscopy. When required, the reaction mixture was taken to pH 3 with 4 M HCl before analysis. In other experiments, aliquots (1.5 mL) of the reaction mixture of Lipo-HT were periodically withdrawn, added with 11 *μ*L of a 10 mM Ellman's reagent solution in 0.1 M phosphate buffer (pH 7.4), and after 15 min the absorbance at 412 nm was read. In other experiments, the reaction was run: (i) in the absence of HgCl_2_ and (ii) under an argon atmosphere. Methanooxocinobenzodioxinone derivatives were obtained by tyrosinase-catalyzed oxidation of HT as previously described [53].

### 2.9. Statistical Analysis

Data are shown as means ± SE. The significance of differences was determined by one-way ANOVA followed by a post hoc Dunnett multiple comparison test with significance set at *p* < 0.05. GraphPad Prism 5 was used for statistical analysis.

## 3. Results and Discussion

### 3.1. Cytoprotective Effects of Lipo-HT and HT on Hg-Induced Oxidative Alterations in RBC

The ability of natural and biobased phenolic antioxidants to counteract Hg-induced cytotoxicity was investigated using olive oil HT and its conjugate with dihydrolipoic acid, Lipo-HT, which in *in vitro* tests proved highly efficient in counteracting the action of ROS and associated cellular damage [48]. Lipo-HT was prepared from tyrosol and dihydrolipoic acid according to a procedure previously developed [48].

Intact RBC were exposed *in vitro* to 40 *μ*M HgCl_2._ Based on our previous results [37] on the dose-dependency of Hg toxic effects in RBC, the concentration of 40 *μ*M was taken as the optimal dose to study the oxidative stress-mediated cytotoxicity in our *in vitro* model. Moreover, it is worth noting that comparable concentrations have been found in the blood of individuals exposed to peculiar working environments like gold mines [16]. Cellular lysis, ROS formation, and intracellular GSH levels were evaluated in RBC following 4 h incubation.

**Figure 2:**
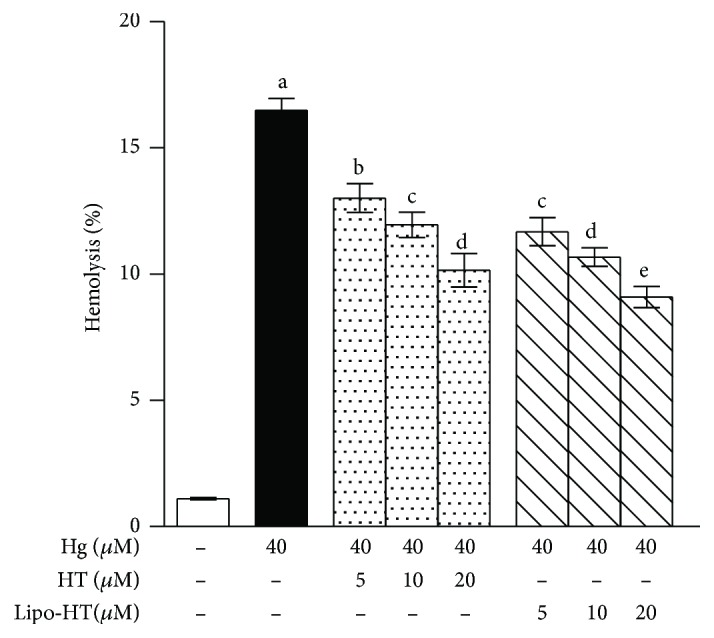
Effect of HT and Lipo-HT on Hg-induced hemolysis. Cells were treated with HgCl_2_ at 40 *μ*M for 24 h in the presence of increasing concentrations of the selected compounds. Data are the means ± SE (*n* = 9). Statistical analysis was performed with one-way ANOVA followed by Dunnett's test (*p* < 0.05). Means with different letters are significantly different.

Prolonged Hg treatment is associated with severe hemolysis; therefore, cell lysis was evaluated by measuring hemoglobin release in the medium upon cell exposure to HgCl_2_. Data in Figure 2 show a dramatic increase in the hemolytic process confirming the cytotoxicity resulting from exposure of cells to HgCl_2_. Both compound Lipo-HT and HT are effective in preventing the toxic effect of the heavy metal as highlighted by the decrease in hemolysis. At all tested concentrations, Lipo-HT shows an enhanced ability over HT on Hg-induced cytotoxicity. Comparable protective effects are provided by Lipo-HT at concentrations halved with respect to those of HT in a statistically significant mode.

**Figure 3:**
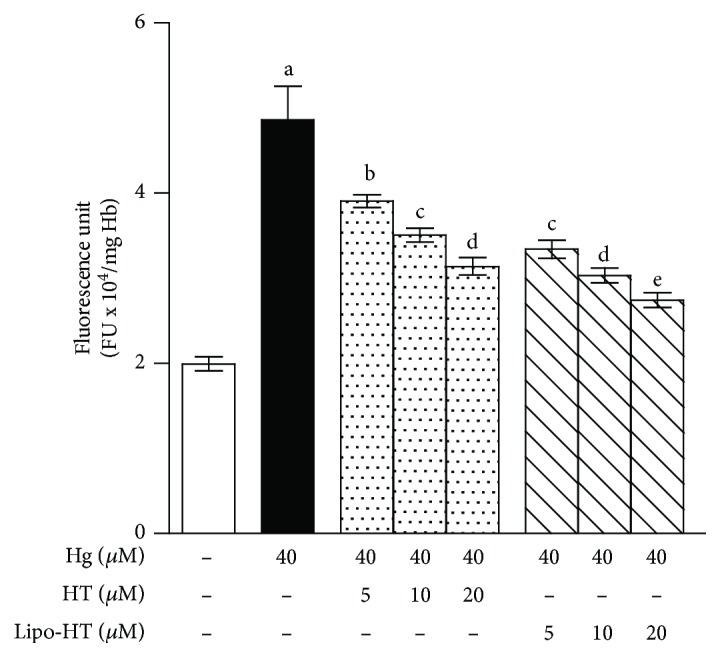
Effect of HT and Lipo-HT on Hg-induced ROS production in RBC. Cells were treated with HgCl_2_ at 40 *μ*M for 4 h in the presence of increasing concentrations of the selected compounds. ROS production was evaluated by means of the fluorescent probe DCF. Data are the means ± SE (*n* = 9). Statistical analysis was performed with one-way ANOVA followed by Dunnett's test (*p* < 0.05). Means with different letters are significantly different.

Figure 3 reports ROS production in the presence of Hg^2+^ and varying amounts of Lipo-HT or HT in the concentration range 5–20 *μ*M as determined by DCF fluorescence assay. The Hg-induced ROS generation is dose-dependently prevented in the presence of increasing concentrations of either Lipo-HT or HT, a significant effect being observable starting from a concentration as low as 5 *μ*M. Interestingly, Lipo-HT appears more effective than HT, producing a 57% decrease of ROS production with respect to the basal level at 5 *μ*M versus an only 34% decrease for HT at the same concentration.

**Figure 4:**
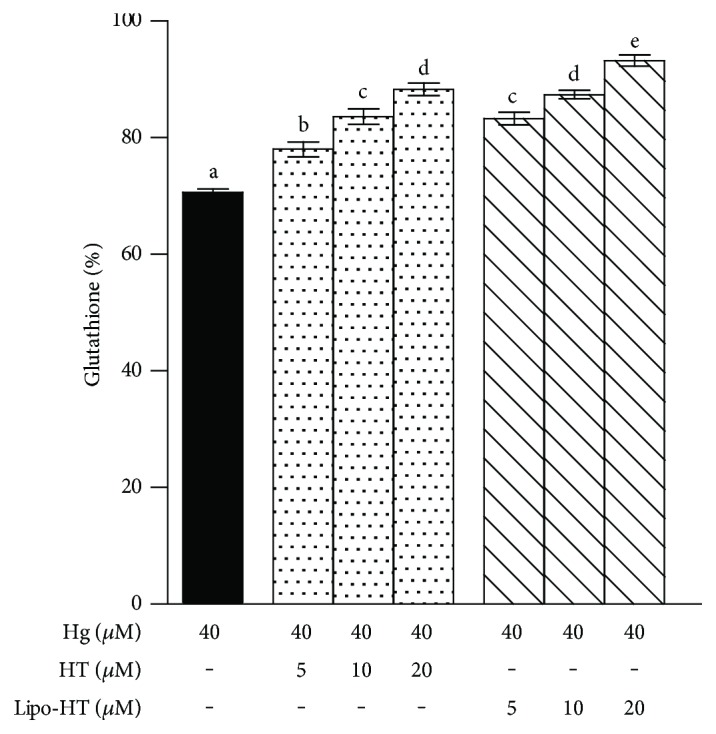
Effect of HT and Lipo-HT on Hg-induced GSH decrease in RBC. Cells were treated with 40 *μ*M HgCl_2_ for 4 h in the presence of increasing concentrations of the selected compounds. Data are the means ± SE (*n* = 9). Statistical analysis was performed with one-way ANOVA followed by Dunnett's test (*p* < 0.05). Means with different letters are significantly different.

To confirm the observed markedly protective action of Lipo-HT on the specific events that ultimately result in Hg toxicity, the effect of the compounds under study on GSH intracellular concentrations was evaluated. Hg^2+^ specifically binds to biological thiols, including GSH, and is able to induce their oxidation with consequent depletion of the intracellular levels. This is generally regarded as a most critical corollary of Hg toxicity, resulting in the impairment of the antioxidant defence system. Data in Figure 4 refer to the levels of GSH in RBC as determined spectrophotometrically by Ellman's assay. Following cell exposure to Hg^2+^, the GSH level is significantly reduced with respect to the control RBC. Also in this case, coincubation with Lipo-HT at 5 *μ*M prevents GSH depletion by about 30%, an effect that is obtained with 10 *μ*M HT.

To assess the role of the lipoyl side chain in the observed protective action of Lipo-HT, in other experiments the effects of lipoic acid on Hg-induced toxicity in RBC were investigated. It is clear that this compound could not fully model dihydrolipoic acid that, however, was not possible to include in the controls due to its high instability. Lipoic acid proved to be quite efficient in controlling the Hg-induced increase of ROS levels with effects at 20 *μ*M statistically comparable to those of HT at the same concentration and those exerted by Lipo-HT at 10 *μ*M. On the other hand, at 5 and 10 *μ*M, lipoic acid did not restore the levels of GSH compared to the control in a statistically significant manner and only at the highest dose tested (20 *μ*M) did it produce effects statistically comparable to those obtained with HT at 5 *μ*M.

### 3.2. Analysis of the Reaction of Lipo-HT and HT with Hg^2+^ Ions at pH 7.4

**Figure 5:**
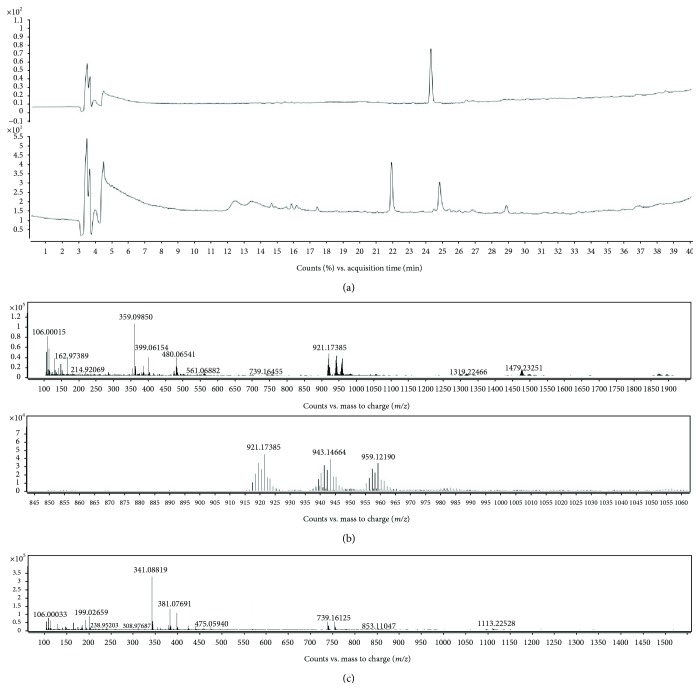
Analysis of the reaction mixture of Lipo-HT with Hg^2+^ ions at pH 7.4. (a) Total ion current (TIC) chromatograms of the reaction mixture of Lipo-HT (50 *μ*M) with Hg^2+^ (100 *μ*M) at pH 7.4 (top: before addition of Hg^2+^, bottom: 5 min after addition of Hg^2+^). (b) Top: MS spectrum of the product eluted at 25 min; bottom: inset showing the Hg isotopic signatures of the complex. (c) MS spectrum of the product eluted at 22 min.

In order to obtain information about the mechanisms responsible for the protective effect of Lipo-HT against the damage induced to RBC by Hg ions, in other experiments the course of the reaction of Lipo-HT (50 *μ*M) with Hg^2+^ (100 *μ*M) at pH 7.4 was followed by HPLC and LC/MS. An almost complete consumption of Lipo-HT was observed after 5 min, with concomitant formation of two major products eluted at about 22 and 25 min (Figure 5(a)). This latter showed pseudomolecular ion peaks [M+H]^+^, [M+Na]^+^, and [M+K]^+^ at *m*/*z* 921, 943, and 959, in that order, suggestive of a 2 : 1 complex of Lipo-HT with Hg. Consistently with the presence of the seven stable isotopes of Hg (with ^202^Hg being the most abundant at 29.86%), these peaks showed a distinct isotopic pattern (Figure 5(b)), which provided further evidence for the formation of the complex [54]. Ellman's assay [55] indicated a more than 90% abatement of sulfhydryl groups in the reaction mixture after 5 min, pointing to the involvement of the thiolate moiety rather than the catechol unit in Hg-complex formation.

**Figure 6:**
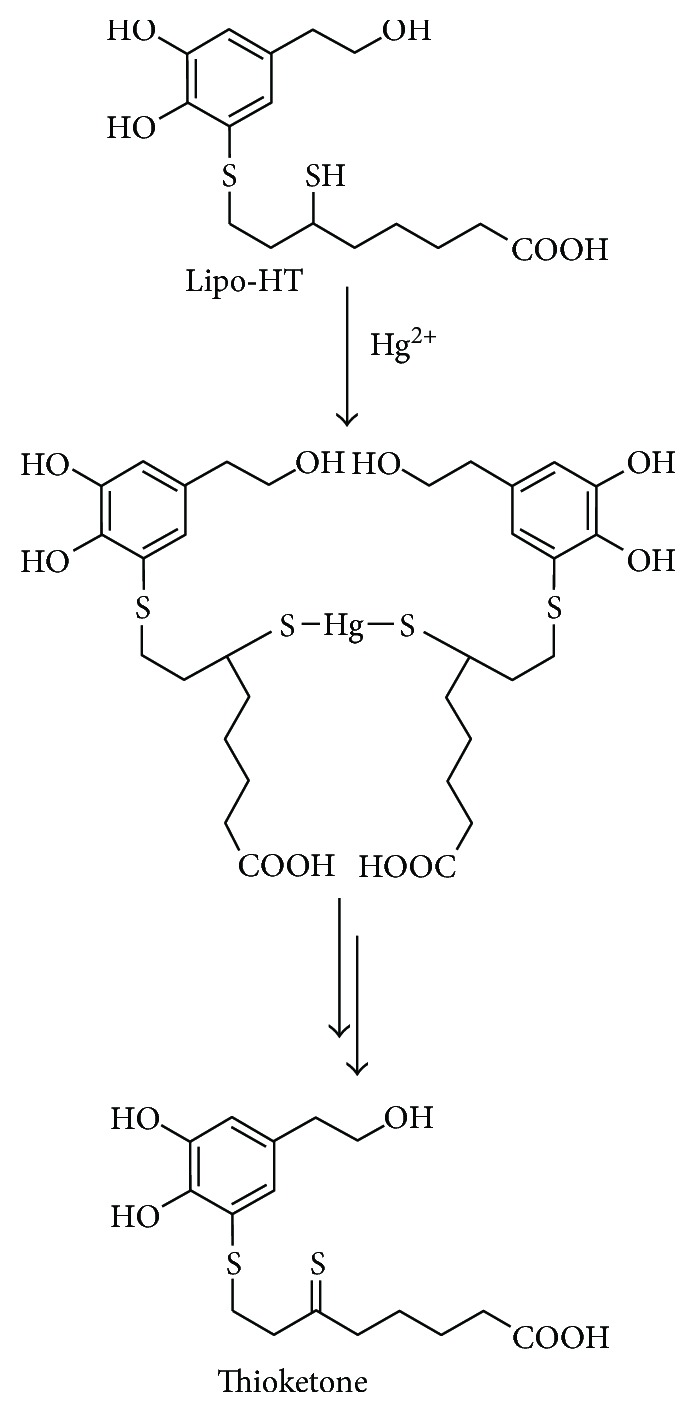
Mechanism proposed for the reaction of Lipo-HT with Hg^2+^ at pH 7.4.

The MS spectrum of the compound eluted at 22 min was characterized by pseudomolecular ion peaks [M+Na]^+^ and [M+K]^+^ at *m/z* 381 and 397, respectively. A [M-H_2_O+H]^+^ at *m/z* 341 together with [2M+Na]^+^ and [3M+K]^+^ at *m/z* 739 and 1113, respectively, were also present (Figure 5(c)). These data were suggestive of an oxidation product of Lipo-HT, likely a thioketone as shown in Figure 6. HPLC and LC/MS analysis of the mixture at 1 h reaction time revealed that the Lipo-HT/Hg complex had disappeared, and it also revealed the presence of thioketone as the only residual product.

In control experiments run in the absence of Hg, only a 20% consumption of Lipo-HT was observed after 1 h, and no other product could be detected, apart from traces of thioketone and of the disulfide of Lipo-HT [48]. Moreover, a complete consumption of Lipo-HT, together with the formation of the products eluted at 22 and 25 min, was observed even when the reaction was run under an argon atmosphere, ruling out a possible role of oxygen in the oxidation reaction.

Based on all these observations, a mechanism for the reaction of Lipo-HT with Hg^2+^ was proposed as depicted in Figure 6. This involves the rapid formation of the Lipo-HT/Hg^2+^ complex, followed by oxidation with formation of a thioketone, likely coupled with the reduction of Hg^2+^. Hence, Lipo-HT would act as both a chelating and a reducing agent toward Hg ions, thus limiting their capacity to induce oxidative damage in biological compartments. Notably, under the same reaction conditions, HT underwent about 30% consumption after 1 h, giving rise to the methanooxocinobenzodioxinone derivatives identified by comparison of the chromatographic behavior and mass spectra with those of authentic samples [53]. LC/MS analysis of the mixture in the early stages of the reaction revealed the formation of an oxidation product of HT, likely *o*-quinone; accordingly, the UV/Vis spectrum of the reaction mixture at the same time showed an absorption maximum at 390 nm, in close agreement with that reported for the *o*-quinone of HT [49]. No Hg complex formation could be observed, either by UV/Vis or by LC/MS analysis.

As to the relevance of the results of this model experiment to the effects observed in RBC, it should be noted that both Hg^2+^ and Lipo-HT and HT concentrations used were higher but comparable to those chosen for the cellular assays. This however does not allow us to extend tout court these findings to the *in vivo* environment where other species occurring at higher levels may compete with Lipo-HT for Hg binding.

## 4. Conclusion

The use of natural compounds of plant origin with a potential to control the oxidative cellular alterations associated with Hg exposure is a strategy that is gaining increasing interest to counteract the toxicity of this and other heavy metal pollutants [56]. In this connection, the present investigation provides a significant contribution showing the potency of a derivative of the olive oil polyphenol HT, which contains the active sulfhydryl moiety of dihydrolipoic acid in the molecular scaffold, in controlling the oxidation events triggered by Hg ions and in protecting the intracellular homeostasis warranted by GSH levels. The effects observed can be ascribed to formation of a Hg complex involving the free secondary SH group followed by a redox reaction that would spare intracellular GSH. It can therefore be concluded that the greater ability of Lipo-HT to protect RBC from Hg-induced damage compared to HT is likely due to the more effective chelating and reducing capacity toward Hg ions, in agreement with the higher reducing power toward iron and copper ions previously reported [48]. Although the catechol moiety of Lipo-HT does not seem to be involved in the Hg complex formation, it may play a role as a scavenger of ROS generated following Hg^2+^-induced metabolic alterations in the cell. Finally, it should be emphasized that the concentrations of HT and its derivative used in this study (5–20 *μ*M) are in the range of plasma concentrations of HT in individuals who adhere to the Mediterranean dietary habit and consume moderate quantities of extra virgin olive oil (25 mL/day). Although further experiments on animal models are clearly needed before the therapeutic use of Lipo-HT against mercury toxicity may be considered, and additional data concerning its absorption, plasma-half-life, and metabolism should be obtained, the results of the present work will expectedly stimulate further studies towards exploitation of this compound in nutraceutical strategies.

## Abbreviations

Lipo-HT: 5-*S*-Lipoylhydroxytyrosol
HT: Hydroxytyrosol
RBC: Red blood cells
ROS: Reactive oxygen species
DCFH-DA: 2′,7′-Dichlorodihydrofluorescein diacetate
Hb: Hemoglobin
DCF: Dichlorofluorescein
DTNB: 5,5′-Dithiobis(2-nitrobenzoic acid) (Ellman's reagent)
TIC: Total ion current
MS: Mass spectrometry.
